# Suppurative dacroadenitis causing ocular sicca syndrome in classic Wegener’s granulomatosis

**DOI:** 10.4103/0301-4738.77044

**Published:** 2011

**Authors:** Dhanita Khanna, Arun Shrivastava

**Affiliations:** Department of Clinical Immunology and Rheumatology, KLES Dr. Prabhakar Kore Hospital and Medical Research Center; and Department of Medicine, JN Medical College, Belgaum, Karnataka, India

**Keywords:** Antineutrophil cytoplasmic antibody, Horner’s syndrome, lacrimal gland, ptosis, Sjogren’s syndrome

## Abstract

Wegener’s granulomatosis (WG) is a multisystem vasculitic disorder which can commonly afflict various components of the eye. Here we describe some unusual ocular manifestations of the disease in one patient. A young male with history of upper respiratory tract symptoms including epistaxis, nasal stuffiness and maxillary sinus pain presented with bilateral lacrimal gland abscess and ptosis. Lacrimal gland biopsy revealed granulomatous vasculitis. Lung cavities, positive cytoplasmic-antineutrophil cytoplasmic antibodies and high titers of serine proteinase-3 antibodies confirmed the diagnosis of WG. The patient developed dry eyes after a month of first presentation. There was no dryness of mouth, suggesting the absence of salivary gland involvement, and antinuclear antibodies as well as antibodies against Ro and La antigens classical of primary Sjogren’s syndrome were absent. Granulomatous vasculitis of lacrimal gland leading to abscess formation and dryness of eyes has not been described in WG and reflects the aggressive nature of inflammatory process in this disease.

Wegener’s granulomatosis (WG) is a systemic vasculitis of unknown etiology. Although it classically involves the upper respiratory tract, lungs and kidneys, virtually any organ system may be affected. Eye involvement is one of the commonest manifestations of WG.[1,2] We describe here some unusual ocular manifestations of the disease in one patient.

## Case Report

**Figure 1:**
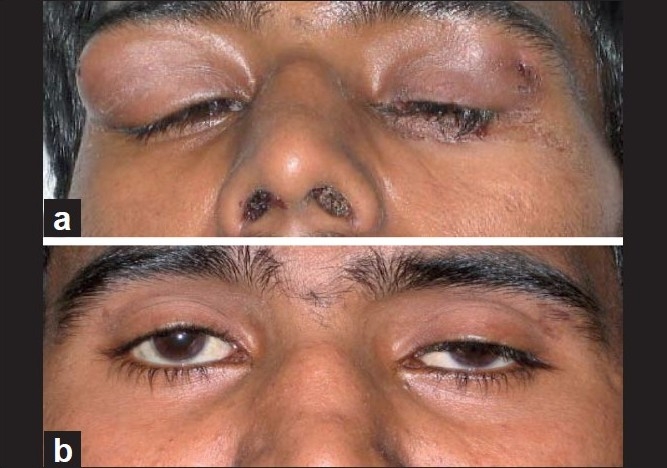
(a) Bilateral dacroadenitis along with nasal crusting; (b) the patient after 1 month of immunosuppressive therapy, showing left-sided ptosis but disappearance of lacrimal gland tumors

**Figure 2:**
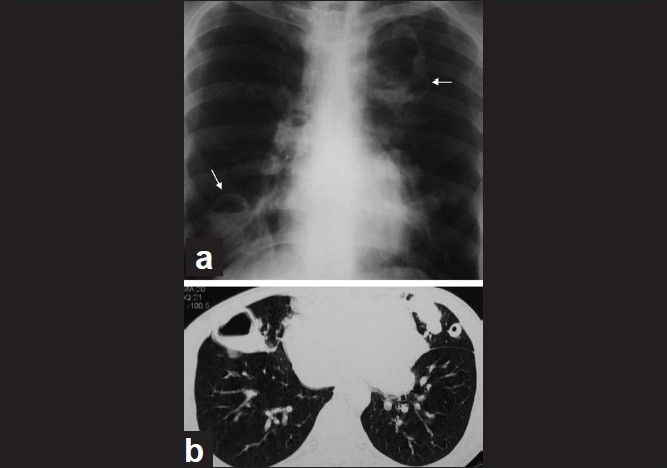
(a) Chest X-ray showing lung opacity and cavities (arrows); (b) CT scan of lungs showing cavities and nodules

**Figure 3:**
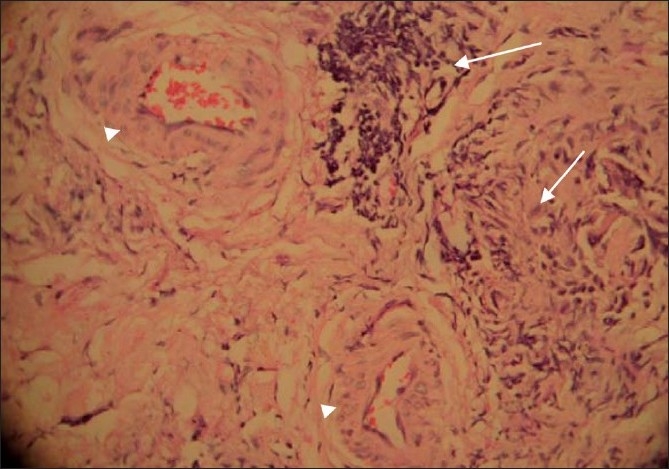
Histopathology of right lacrimal gland tissue showing necrotizing vasculitis (arrowheads) and organizing granuloma (arrow)

A 27-year-old man complained of 2-month history of nasal stuffiness, epistaxis and maxillary sinus pain. One month later, he developed painful swellings over the outer part of both eyes. He was diagnosed elsewhere as presumed lid abscess and managed with antibiotics (amoxicillin-clavulanic acid) and drainage of purulent material from these swellings. He presented to us after another month with recurrence of eye swelling along with new development of dry cough. There was no history of redness of eyes, blurring of vision, diplopia, dry eyes, hemoptysis, dyspnea, fever, oliguria, hematuria, skin rash, tingling, burning or weakness in extremities, headache, seizures or altered sensorium. Examination revealed bilateral S-shaped swelling diagnosed as lacrimal gland swelling [Fig. 1a] by our ophthalmologist. There was bilateral ptosis but no ophthalmoplegia or Horner’s syndrome. There was no uveitis or retinal involvement. The patient was normotensive and systemic examination was unremarkable. Investigations revealed polymorphonuclear leukocytosis and thrombocytosis. Complement proteins C3, C4 and renal functions were normal. Urine examination revealed active sediments [white blood cells (WBC) 20-25/high power field (HPF), red blood cells (RBC) 6-8/HPF], but no albuminuria. Chest radiography and computerized tomography (CT) scan showed nodular and cavitating lesions [Fig. 2a, b]. CT orbit did not reveal any retro-orbital mass. Culture of pus from past incisions over lacrimal gland did not reveal any organism. Incisional biopsy done from lateral aspect of left lacrimal gland swelling showed fibro-areolar tissue with necrotizing granulomatous vasculitis[Fig. 3]. Positive cytoplasmic-antineutrophil cytoplasmic antibody (c-ANCA) and high serine proteinase-3 levels (366 U/ml; normal <16 U/ml) confirmed the diagnosis of WG. The patient was treated with initial regimen of daily oral cyclophosphamide (2 mg/kg) and oral prednisolone (1 mg/kg) which was gradually tapered after initial remission was obtained.

A month later, there was complete regression of lacrimal gland swelling, but left-sided ptosis [Fig. 1b] continued to persist. He also developed marked dryness of eyes and Schirmer’s I test was positive (2 mm wetting in both the eyes after 5 minutes). There was no dry mouth, diplopia or ophthalmoplegia. Antinuclear, anti Ro and anti La antibodies were negative. There was complete regression of lung cavities with the above treatment after 3 months. After 1 year of follow-up, the patient was in complete remission with no recurrence of lacrimal gland swelling or lung lesions. However, ptosis of the left eye and ocular dryness continued to persist and the patient required frequent instillations of artificial tear drops.

## Discussion

Ocular involvement is one of the commonest features of WG and may be seen in 30-50% of patients.[1,2] It may precede,[3,4] or follow,[4] the other classic manifestations of WG. Rarely, patients may present only with ocular disease without systemic involvement.[5] Ocular manifestations of WG can occur as a result of contiguous granulomatous sinusitis (naso-lacrimal duct obstruction, proptosis, ocular muscle or optic nerve involvement) or isolated focal vasculitis (pseudo-tumor orbitae, conjunctivitis, episcleritis, scleritis, corneo-scleral ulceration, uveitis, and granulomatous vasculitis).[2] Isolated or predominant lacrimal gland involvement and ptosis in WG has been uncommonly reported, while suppurative dacroadenitis leading to ocular sicca syndrome has not been described.

Although lacrimal gland involvement leading to dry eyes is commonly seen in autoimmune connective tissue disorders, dacroadenitis is uncommon in most, except primary Sjogren’s syndrome. In WG, it may be sole presentation of the disease with absence of systemic symptoms,[5] or may be an early or first manifestation of classic WG followed by other extra-ocular features,[6–8] as also in our patient, and should warrant a search for other features of WG. Although multifocal vasculitis leading to aggressive ocular syndrome is known, our patient had predominantly lacrimal gland disease which did not progress beyond the lacrimal glands. We feel the patient lacked other common ocular manifestations since he was diagnosed early in the disease course leading to therapeutic intervention.

Lacrimal gland abscess has only been reported once in literature where it was associated with infection,[9] but has not been reported in vasculitides. Our patient had significant dryness of eyes within a few weeks of the lacrimal gland involvement. We attribute this to extensive lacrimal gland necrosis in view of the absence of features to suggest the possibility of secondary Sjogren’s syndrome. Possibility of iatrogenic incision related lacrimal ductile damage or a sequel to lacrimal gland biopsy did not appear the likely cause for dry eyes in view of significant time lag between these procedures and development of dry eyes. To the best of our knowledge, lacrimal gland abscess leading to ocular sicca syndrome has not been reported in the English literature. Even in the cases of infective abscess as reported earlier,[9] neither of the patients went on to develop dry eyes.

Ptosis is relatively a common feature in large vessel vasculitides, such as temporal arteritis and Behcet’s disease, but has rarely been described in WG.[10] While in large vessel vasculitis oculomotor nerve involvement is the most likely cause, in WG there may be multiple ocular pathologies, including retinal involvement. Our patient did not have any other ocular features except dacroadentis. While right eye ptosis improved with regression of lacrimal gland tumor suggesting mechanical ptosis, left-sided ptosis persisted despite regression of lacrimal gland swelling. We postulate that the ptosis in our patient was probably due to focal angiopathy and ischemia leading to partial sympathetic nerve involvement. A possibility of iatrogenic complication subsequent to lacrimal gland biopsy causing dehiscence of levator palpabre superioris, however, could not be ruled out.

Thus, this was a rare case with uncommon ocular manifestations of WG in one patient. Dacroadenitis in WG can be one of the early features of the disease and may be associated with poor ocular outcome including suppurative dacroadenitis and dry eyes.
